# Premature ventricular contractions: pathophysiology, prognostic impact, and management

**DOI:** 10.1093/ehjopen/oeag164

**Published:** 2026-10-13

**Authors:** Raffaele Scorza, Pasquale Santangeli

**Affiliations:** Department of Cardiology, Södersjukhuset, Sjukhusbacke 10, Stockholm 118 83, Sweden; Center for Heart Failure and Arrhythmia, Karolinska Institutet, Stockholm, Sweden, Sjukhusbacke 10, Stockholm 118 83, Sweden; Cleveland Clonic Foundation, 500 Euclid Ave., Cleveland, Ohio 44195, USA

**Keywords:** Ventricular, Premature, Complexes, Arrhythmia, Evaluation, Ablation

## Abstract

Premature ventricular contractions (PVCs) arise from an early depolarization originating in either of the cardiac ventricles. They are common in individuals both with and without structural heart disease, although their true prevalence remains uncertain. Several electrophysiological mechanisms can initiate the PVC, with re-entry being the most frequent. The detection of PVCs should prompt a thorough clinical evaluation to identify potential underlying cardiac disease, yet many PVCs ultimately prove to be idiopathic. In individuals with established structural heart disease, PVCs have been associated with a poorer prognosis; however, clinical trials aimed at suppressing PVCs in this population have not demonstrated improved outcomes. In contrast, the prognostic significance of PVCs in patients without structural heart disease remains unclear. Factors such as PVC burden, PVC morphology, and cardiac magnetic resonance findings have been proposed as prognostic markers, although the evidence is still debated. In general, patients without structural heart disease and with a low PVC burden can be reassured regarding their prognosis. Treatment is primarily guided by symptoms, although reducing PVC burden may be considered even in asymptomatic individuals to mitigate the risk of complications such as PVC-induced cardiomyopathy or PVC-triggered sustained ventricular arrhythmias. Antiarrhythmic drugs are commonly used as first-line therapy, and the choice of medication should take into account patient characteristics, suspected pathophysiology, and PVC features. Catheter ablation (CA) is more effective than pharmacologic therapy in reducing or eliminating PVCs and may be considered as a first-line option in specific situations where rapid PVC suppression is desired. As an invasive procedure, CA carries potential risks that must be weighed carefully. Operator experience, PVC origin, anatomical considerations, and patient preference all play important roles in decision-making. Further research is needed to better identify patients at risk and to target specific pathophysiological mechanisms. Advances in device-based, patient-driven arrhythmia monitoring and the integration of artificial intelligence are likely to influence future PVC diagnostics and risk stratification.

## Graphical Abstract

**Graphical Abstract:**
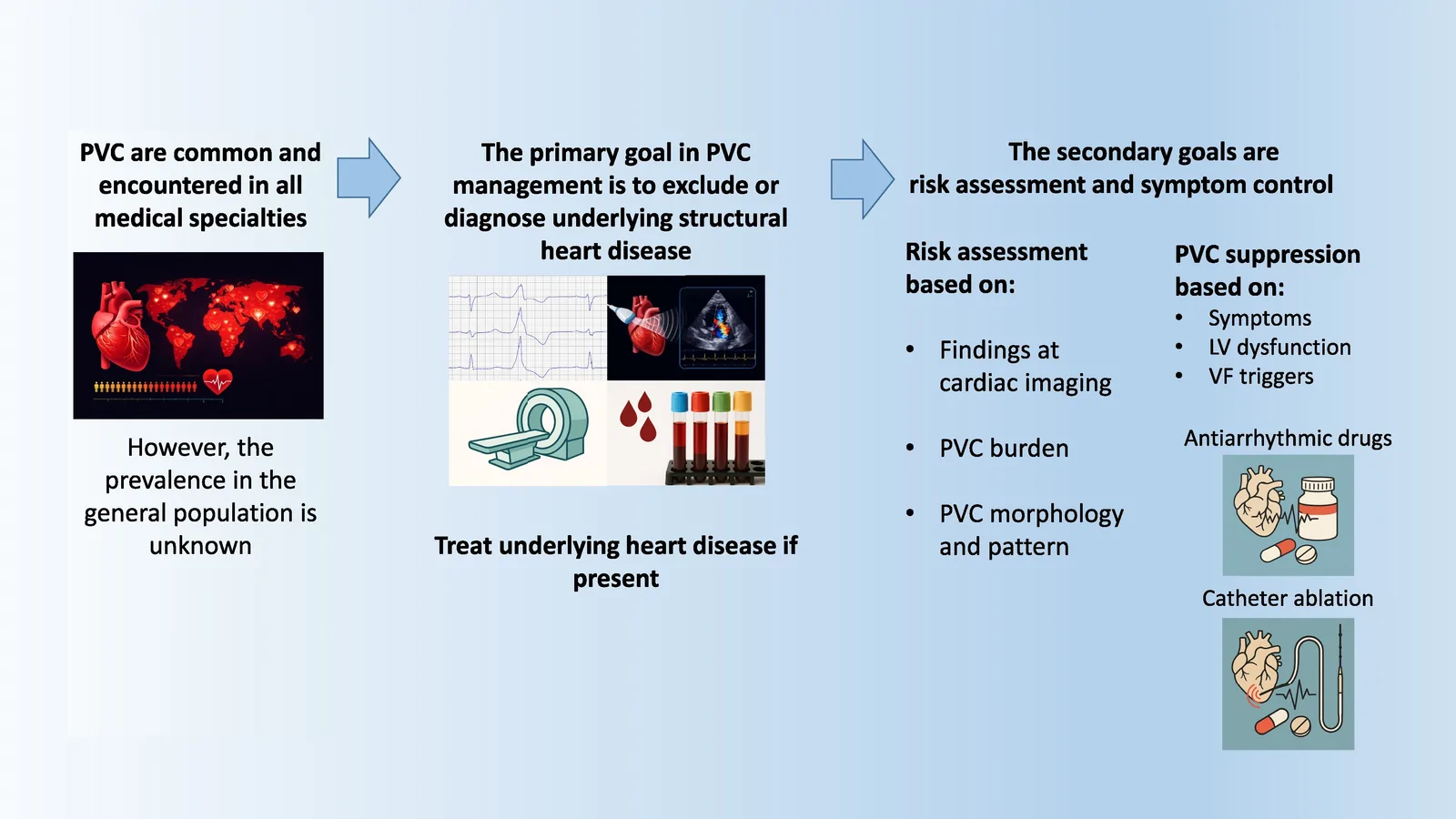

## Introduction

Premature ventricular complexes (PVCs) are early ectopic beats originating from either of the two ventricles. They were described already in the 18th century^1^ and are most commonly identified on a 12-lead electrocardiogram (ECG), appearing as a widened QRS complex (typically >120 milliseconds) that occurs earlier than the expected normal complex and without a preceding P wave. PVCs are generally followed by a compensatory pause and are associated with a discordant ST-T segment. The site of origin can be inferred from the morphology on the 12-lead ECG, while PVCs may be further classified according to frequency (expressed as absolute counts or percentage of total beats/PVC burden), coupling interval, QRS duration, and complexity (unifocal, multifocal, couplets, triplets). Alternative terminology includes premature ventricular beats, premature ventricular depolarizations, premature ventricular contractions, ventricular ectopic activity, ventricular ectopic beats, ventricular premature depolarizations, and ventricular extrasystoles. Nevertheless, the 2019 HRS/EHRA/APHRS/LAHRS consensus statement on catheter ablation of ventricular arrhythmias consistently uses the term *premature ventricular complexes (PVCs)*, and this terminology has subsequently been widely adopted in the literature.^2^ This terminology emphasizes that the electrical activity of a PVC does not invariably result in a mechanical ventricular contraction.

PVCs are frequently encountered in clinical practice and may occur in individuals both with and without structural heart disease (SHD).^3^ In patients without evidence of structural or inherited cardiac pathology, PVCs are classified as idiopathic. The predominant source of idiopathic PVCs is the right ventricular outflow tract (RVOT)^4^ with left ventricular outflow tract and intracavitary ventricular structures following in frequency. Other sites of origin, such as the valvular annular regions, the left bundle fascicles or epicardial foci, are more uncommon.^5^ PVCs may be entirely asymptomatic and discovered incidentally, or they may manifest with symptoms that range from bothersome to debilitating, such as palpitations—often perceived as chest discomfort, irregular heartbeats, or the sensation of a missed beat—along with dizziness, dyspnoea, or fatigue.^6^ Determining whether PVCs in patients without SHD are truly idiopathic, and whether they influence prognosis, is of considerable clinical importance, as it directly informs decisions regarding diagnostic evaluation, follow-up, and therapeutic strategies.

## Epidemiology

Numerous studies have investigated the PVC prevalence in the general population, employing diverse inclusion criteria and varying approaches to electrocardiographic recording, which has resulted in markedly heterogeneous findings. One of the earliest large-scale ECG-based epidemiological investigations was conducted in 1962, when Roland Hiss and Lawrence Lamb examined approximately 120 000 apparently healthy males and reported that nearly 5% exhibited electrocardiographic anomalies, with 7.8 per 1000 demonstrating PVCs.^7^ Data from the ARIC study indicate a PVC prevalence of about 5%, rising to 23% among subjects with hypertension.^8^ Fisher reported an incidence of about 7%,^9^ Kennedy et al. estimated a prevalence of 1–4% in the general population,^10^ while Kostis *et al*. reported a prevalence approaching 40%.^11^ The MRFIT study,^12^ based on a large cohort, reported a prevalence of 4%, while Yang *et al*., in another large cohort undergoing Holter monitoring, found that 1.3% of the patients had a very high burden of PVCs (≥20% of total heartbeats).^13^ More recently, patch-based ECG has enabled extended recording periods, and screening of an elderly population revealed that 99.5% experienced at least one isolated PVC per hour, with 34.6% demonstrating ventricular tachycardia (defined as more than four consecutive ventricular beats) over a 24-hour period.^14^ Some major studies investigating PVC prevalence in population-based cohorts or healthy individuals are summarized in *Table 1*.

**Table 1:** Summary of landmark studies investigating PVC prevalence

First author	Publication	Population	Screening technique	Approximate Prevalence
Hiss RG^7^	Circulation, 1962	122 043 apparently healthy males (mostly US Air Force personnel)	ECG	1%
Fisher FD^9^	Circulation, 1973	1 214 factory employees	ECG	7%
Kostis JB^11^	Circulation, 1981	101 individuals with normal results at comprehensive examination	24 h ambulatory ECG	40%
Abdalla ISH^12^	The American Journal of Cardiology, 1987	Community-based cohort of apparently healthy 15 637 individuals	2-minute lead I rhythm strip	4%
Simpson RJ^8^	American Heart Journal, 2002	Community-based cohort of 15 792 individuals	2-minute ECG	6%

## Pathophysiology

Three principal mechanisms have been implicated in the genesis of ectopic beats: re-entry, increased automaticity, and triggered activity. For a re-entry phenomenon to occur, two distinct electrical pathways must be present, with one pathway exhibiting unidirectional block. These pathways provide the substrate for arrhythmogenesis and may involve either established anatomical structures (e.g. bundle branches, fascicles) or regions with altered conduction velocity resulting from pathological processes in cardiac tissue. A classic example is the fibrotic scar produced by myocardial infarction or myocarditis. When a depolarization wave traverses such a fibrotic area, conduction may be sufficiently delayed to reach adjacent healthy myocardium that is no longer refractory, thereby initiating one or more ectopic beats. This mechanism helps explain the higher prevalence of PVCs in populations with increased cardiovascular risk, including males, smokers, the elderly, and individuals with hypertension or impaired ventricular function.^8,15,16^ Triggered activity occurs when excessive calcium influx elevates intracellular concentrations, leading to spontaneous calcium release from the sarcoplasmic reticulum and subsequent delayed afterdepolarization. However, the precise mechanisms by which this process generates focal electrical activity remain incompletely understood.^17^ Prolonged repolarization and delayed afterdepolarization are observed under conditions such as bradycardia, digitalis intoxication, hypokalaemia, and Long QT syndrome.^18–20^ Enhanced electrical automaticity—defined as the intrinsic ability of myocardial cells to depolarize spontaneously and initiate new depolarization waves—may also contribute to PVC formation. This mechanism is multifactorial, encompassing catecholaminergic stimulation, transient or permanent tissue alterations, and inherent cellular properties.^21,22^ Although these mechanisms may seem primarily of academic interest, understanding the biological basis of PVCs is clinically significant, as it represents a key determinant of therapeutic success.^23^

The role of mitral valve prolapse (MVP) in PVC pathophysiology deserves a special mention. Up to 1–3% of people have MVP, and this condition carries a connection to PVCs, which are seen in about 40–50% of affected individuals^24^ The underlying mechanism is thought to involve the prolapsing valve leaflet physically tugging on the papillary muscles; this mechanical stress can activate stretch-sensitive afterdepolarizations and trigger abnormal automaticity in the distal Purkinje network.^25^ Adding to this, heightened sympathetic activity observed in some of these patients tends to worsen the degree of ventricular ectopy. For the most part, PVCs linked to MVP carry a benign prognosis. However, when these ectopic beats become complex or pleomorphic, particularly if they originate from Purkinje tissue, they can potentially set off ventricular fibrillation and lead to sudden cardiac death. This risk climbs notably in patients who also show bileaflet prolapse, mitral annular disjunction, or fibrotic changes in the myocardium detected via cardiac magnetic resonance imaging (CMR).^26^

## Diagnostic workup

Frequent premature ventricular contractions (PVCs) are strongly associated with a range of comorbid conditions; therefore, clinical evaluation should primarily focus on identifying or excluding underlying cardiovascular and non-cardiovascular disease. A comprehensive medical history is indispensable, encompassing presenting symptoms, precipitating factors, established cardiac pathology, cardiovascular risk profiles, current pharmacotherapy, prior medical conditions, and relevant family history. In addition to a standard physical examination, diagnostic assessment should include a 12-lead electrocardiogram (ECG) to delineate PVC morphology and to detect potential markers of concomitant cardiac or electrolyte disturbances. Laboratory investigations should evaluate serum electrolytes (potassium, magnesium, and calcium), renal function, and thyroid-stimulating hormone levels. For patients whose symptoms are exercise-induced, an exercise stress test is indicated. Furthermore, ambulatory ECG monitoring should be utilized to quantify PVC burden and to establish correlations between arrhythmic events and patient-reported symptoms.^27,28^ Echocardiography is widely recognized as the first-line imaging modality, providing critical information on cardiac function and the detection of potential structural abnormalities.^27,29^ Clinical evaluation should also encompass the exclusion of underlying coronary artery disease, guided by the patient’s estimated pretest probability. When SHD other than coronary artery disease is suspected based on initial findings, CMR should be considered as a complementary diagnostic tool.^28,29^ In recent years, the role of CMR has been increasingly emphasized, particularly given that the detection of fibrosis by CMR has been associated with poorer prognostic outcomes in this patient population.^30^ Given that CMR is a resource-intensive examination, efforts have been made to delineate and standardize its indications in patients with premature ventricular contractions (PVCs).^31^ A suggested diagnostic work-up is described in *Figure 1*.^7–9,11,12^

**Figure 1:**
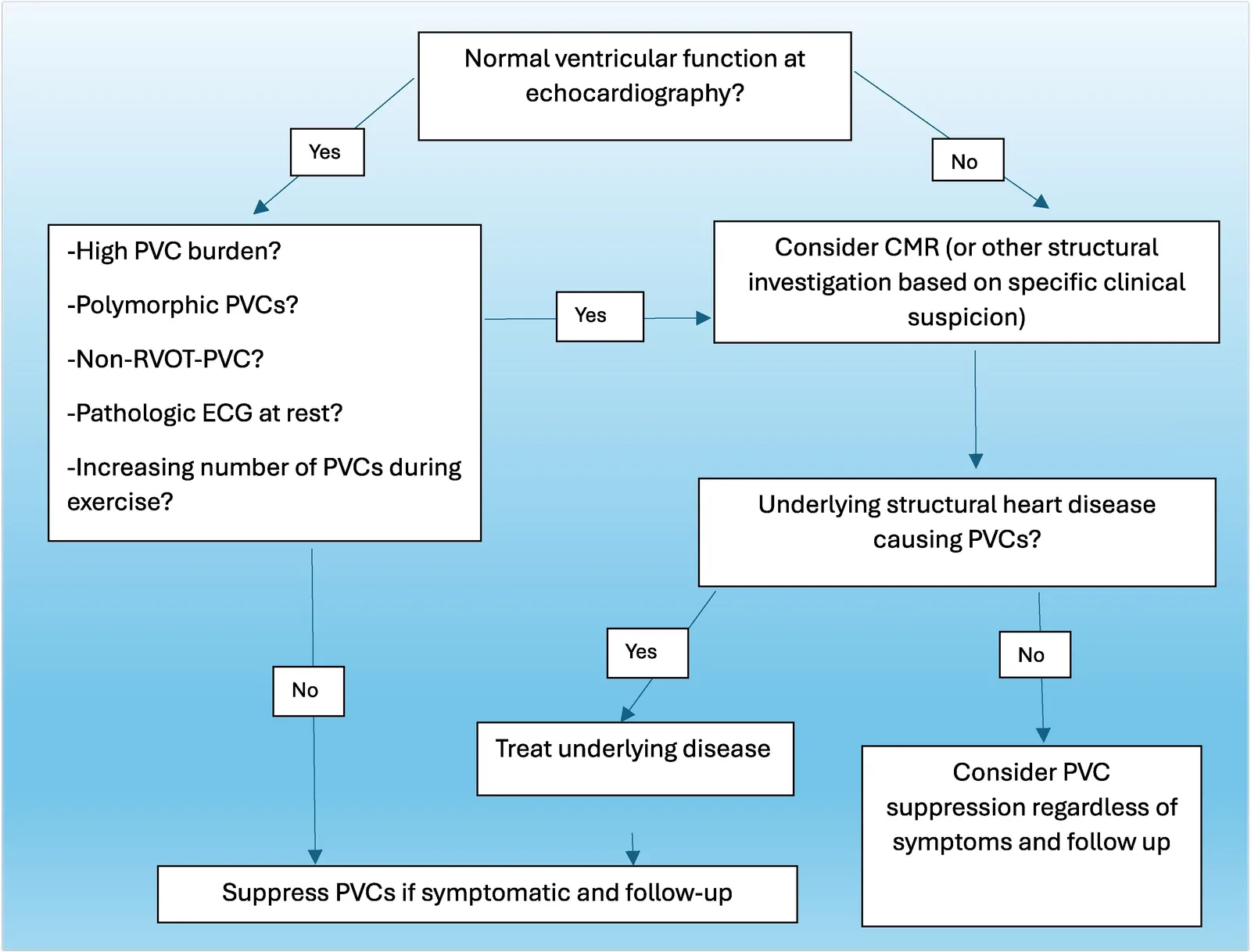
Suggested clinical work-up for PVC patients.

## Prognostic impact—historic perspective of the evidence

In 1985, Kennedy *s*. published a study of 73 asymptomatic, healthy individuals with frequent and complex ventricular ectopy who had been followed for 10 years, demonstrating prognoses comparable to those of the general population.^10^ These findings were confirmed in a subsequent study by the same author^32^ underscoring the continued interest in clarifying the prognostic implications of PVCs in healthy subjects.^33^ Numerous studies published throughout the 1980s and 1990s produced conflicting results. A common criticism of these investigations was the insufficient application of rigorous diagnostic methods to exclude underlying cardiac disease.^34^ In 1992, Kostis summarized the available evidence, concluding that PVCs do not independently contribute to mortality but may instead serve as markers of occult heart disease.^33^ The adverse prognostic impact of PVCs is more evident in the presence of structural heart disease. Findings from studies conducted in the 1960s and 1970s were corroborated in subsequent decades, particularly among patients with prior ischaemic events.^35–38^ Even in non-ischaemic cardiomyopathy, a relationship between PVCs and poor outcomes has been established.^39,40^ In a 1993 review,^41^ Myerburg *et al*. emphasized the role of PVCs in the pathophysiology of malignant arrhythmias, identifying them as the link between ischaemia and ventricular tachycardia/fibrillation. Meanwhile, the CAST trial demonstrated that suppression of ventricular ectopy with class I antiarrhythmic drugs in myocardial infarction survivors did not improve prognosis; on the contrary, patients treated with flecainide or encainide experienced higher mortality compared to those receiving placebo.^42^ These findings reinforced the conclusion that PVCs are not independent contributors to mortality but rather markers of underlying disease. While the prognostic impact of PVCs in structural heart disease appears indisputable, evidence regarding their role in healthy individuals remains uncertain. Many studies reporting a negative impact relied solely on patient history to exclude structural disease and/or were conducted exclusively in male populations^12,43–48^. Among large population studies, Agarwal^49^ demonstrated a higher incidence of heart failure (HF) in PVC patients compared with controls. Notably, the association between PVCs and incident HF was strongest in patients with fewer cardiovascular risk factors, with a six-fold increase in risk among those younger than 65 years without CHD, diabetes, hypertension, or atrial fibrillation. In a 12-year follow-up of Japanese men and women,^50^ Hirose reported increased cardiovascular mortality among men with PVCs, whereas no prognostic impact was observed in women. More recent studies in Asian populations have again shown associations between PVCs and all-cause mortality, hospitalization, and HF at the population level.^48,51^ Among the studies that incorporated echocardiographic evaluation at baseline, Dukes *et al*.^52^ examined 1139 individuals whose PVC frequency was quantified by Holter monitoring. They found that individuals in the highest quartile of PVC frequency had a threefold risk of a five-year decline in LVEF, a 48% increased risk of incident CHF, and a 31% increased risk of death compared to those in the lowest quartile, after adjustment for confounders and a median follow-up of 13 years. Results were similar when PVCs were analysed as a continuous variable. In contrast, Lee et al^53^ reported that only a small proportion of PVC patients with normal LVEF developed systolic dysfunction during approximately 5 years of follow-up.

Alongside studies demonstrating a negative prognostic impact of PVCs in the general population or in cohorts without apparent heart disease, others found no such association. Fisher^9^ reported that the risk of coronary artery disease was not increased by the presence of PVCs on routine ECG in a cohort of factory workers aged 35–69. A decade later, Kennedy et al^10^ published results from a 6.5-year follow-up of 73 asymptomatic, healthy subjects with frequent ventricular ectopy, reporting only one case of sudden death. Compared to the standardized mortality ratio, this suggested that long-term prognosis was not adversely affected by PVCs. This study contributed significantly to the view that PVCs are benign in the absence of structural heart disease, and several subsequent publications appear to confirm its findings.^53–58^ In a study specifically focused on the potential development of Arrhythmogenic Right Ventricular Cardiomyopathy (ARVC), Gaita et al^59^ followed 61 patients with frequent right-ventricular PVCs. After an average follow-up of 15 years, no patient had died of SCD or developed ARVC, and half no longer exhibited PVCs. In a cohort of 5000 athletes, Verdile et al^60^ observed that exercise-induced PVCs tended to resolve spontaneously or could be successfully treated, without affecting clinical outcomes.

Although studies reporting a negative prognostic impact of PVCs in subjects without apparent heart disease outnumber those showing no association, the overall evidence remains divided and inconclusive. This uncertainty is compounded by the fact that few studies consistently employed imaging diagnostics to exclude structural disease, relying instead on patient history. Meta-analyses addressing this issue were lacking until 2012, when Lee et al^61^ published the first, followed shortly by Ataklte and colleagues.^62^ Lee’s meta-analysis included studies with control populations based on adults without apparent heart disease, excluding unselected populations not evaluated for structural disease, studies of exercise-induced PVCs, and subgroup analyses within PVC cohorts. When multiple studies were based on the same cohort, only the most recent was included. Out of more than 3000 studies published between 1966 and 2011, only eight met these criteria, all of which were prospective observational cohort studies. Sensitivity analysis revealed that negative prognostic effects were observed primarily in male-only cohorts, while the single study incorporating echocardiography and exercise testing at baseline did not demonstrate worsened outcomes. According to the authors, heterogeneity among studies was substantial, and publication bias was not apparent. The pooled OR for death, cardiovascular death, SCD, or ischaemic heart disease was 1.72 (95% CI 1.28–2.31). Classic cardiovascular risk factors such as male gender, age, diabetes, and hypertension increased the odds of cardiovascular events, suggesting that patients with undetected structural disease were included, thereby influencing results. The authors concluded that if more advanced diagnostic techniques had been used to exclude structural disease, benign outcomes with PVCs would likely have been observed. Consistent with this, study quality assessment indicated that the increase in OR was less pronounced in higher-quality studies. Ataklte *et al*. included prospective cohort, nested case-control, and retrospective cohort studies, ultimately analysing 11 relevant investigations involving 106 000 patients. The prevalence of PVCs ranged from 1.2% to 10.7%, with mean follow-up times of 5–30 years. The six prospective studies showed no significant heterogeneity. The pooled RR for total cardiac death was 2.1 and the authors proposed two hypotheses: PVCs may trigger fatal ventricular arrhythmias, or they may represent markers of underlying disease (reverse causation). They also noted insufficient adjustment for confounders in some studies, though they did not emphasize the lack of diagnostic imaging as strongly as Lee et al.

More recent publications have continued to present conflicting evidence, with some studies suggesting a benign clinical course,^53,57,58^ whereas others indicate a negative prognostic impact.^48,63^ Two review articles recommend that patients with a low PVC burden and normal echocardiographic findings may be reassured, while those with a potentially significant burden or deemed at risk for cardiomyopathy should undergo regular clinical follow-up.^64,65^ In a Japanese study based on retrospective data from an administrative database,^66^ the frequency of PVCs, based on Holter monitoring, was not associated with clinical events, while the presence of non-sustained ventricular tachycardia was independently associated to heart failure incidence. A recent third meta-analysis, encompassing 25 studies, examined the relationship between PVCs and cardiovascular outcomes in populations without overt structural heart disease.^67^ The analysis demonstrated that frequent PVCs were significantly associated with an increased risk of mortality, heart failure, atrial fibrillation, and stroke. However, the authors emphasized that, according to the GRADE framework, the overall quality of evidence was rated as low. This assessment was primarily attributed to the observational nature of the included studies and methodological limitations, such as incomplete adjustment for clinically relevant confounders related to underlying heart disease. Additionally, concerns regarding residual confounding and selection bias, which may restrict the generalizability of the findings, were highlighted as further limitations. Some of the more recent studies investigating the prognostic impact of PVCs in healthy or apparently healthy subjects are summarized in *Table 2*.

**Table 2:** Summary of some of the studies published in the last fifteen years exploring prognostic significance of PVCs in healthy patients

First author	Publ. year	Design/population	Main findings
Lee^68^	2012	Meta-analysis of eight studies with a total of 37 387 patient years followed	PVC patients had an increased risk for all cause-mortality when compared to PVC-free individuals. Most included studies did not have sufficient methods to exclude concomitant heart disease.
Ataklte^62^	2013	Meta-analysis of eleven studies comprising a total of 106 195 participants	Increased risk of mortality was shown for PVC patients. The included studies had no significant heterogeneity and were population-based, implying a high external validity.
Dukes^52^	2015	Follow-up of 1 139 individuals over 65 years of age examined with 24-hour ambulatory ECG and echocardiography	Correlation between number of PVCs and risk for developing LV dysfunction.
Agarwal^49^	2017	California healthcare-data-based comparison of 35 817 subjects with PVC diagnosis to 16 million without PVC diagnosis	Increased risk for incident systolic heart failure after appropriate adjustment.
Lee^64^	2019	Follow-up of 100 individuals with >5% PVC burden and normal echocardiography	A few patients developed LV dysfunction.
Nomura^57^	2020	Student screening of 82 000 first graders and 87 000 seventh graders	Individuals with PVC showed a generally favourable course.
Ogiso^66^	2025	6 332 patients without structural heart disease from a hospital database	The frequency of PVC was not associated with mortality or heart failure. The presence of non-sustained ventricular arrhythmia was associated with a higher risk of heart failure hospitalization

Meta-analyses have also examined exercise-induced PVCs. Kim et al,^69^ analysed nine studies including 62 000 participants, finding that PVCs during recovery were associated with increased risk of death (RR 1.55, 95% CI 1.22–1.96), whereas PVCs during exercise did not reach statistical significance (RR 1.14, 95% CI 0.96–1.34). However, overall effects during exercise testing corresponded to an RR of 1.41 (95% CI 1.23–1.61) for all-cause mortality and 1.86 (95% CI 1.51–2.30) for cardiovascular mortality. Similar conclusions were reached by Lee *et al*.^70^ in 2017, who found that only PVCs during recovery, not during exercise, were associated with poor prognosis. The authors suggested autonomic dysregulation,^71,72^ with increased sympathetic drive creating a pro-arrhythmic substrate, as a possible mechanism leading to fatal outcomes.

Extra-cardiac clinical outcomes have also been investigated in relation to PVCs. Studies from Ofoma^73^ and Agarwal^74^ demonstrated an increased risk of ischaemic stroke among individuals with PVCs. A plausible explanation for this association is atrial fibrillation (AF), as the risk of developing AF has been shown to rise with increasing PVC incidence.^75^ However, in studies where underlying structural disease was carefully excluded from the PVC population, no correlation with ischaemic stroke was observed.^76^ Even in the absence of a prior diagnosis of stroke or transient ischaemic attack, central neurological symptoms have been linked to a high PVC burden (>10%).^77^

## PVC-induced cardiomyopathy

When summarizing the evidence regarding the prognostic impact of PVCs in subjects without heart disease, PVC-induced cardiomyopathy (PVC-CM) must be considered separately since this entity, first described approximately 30 years ago, has a distinct pathophysiology. The earliest reports of PVC-CM were published in 1997 by Vijgen^78^ and in 2000 by Chugh.^79^ While Vijgen and colleagues described a patient with repetitive non-sustained ventricular tachycardia and dilated cardiomyopathy, in whom ablation of the arrhythmia focus led to recovery of ventricular systolic function,^78^ Chugh’s paper was the first to directly associate cardiomyopathy with isolated, frequent PVCs, documenting recovery from heart failure after successful radiofrequency ablation. A few years later, Shiraishi published a similar case report^80^ and recommended ablation as the treatment of choice in such patients. The pathophysiology of PVC-CM is likely distinct from the multiple mechanisms implicated in the broader impact of PVCs summarized thus far. Evidence for this distinction lies in the reversibility of PVC-CM, which is associated with a favourable prognosis when PVCs are effectively treated. It is possible that PVC-CM historically contributed to the general prognostic impact attributed to PVCs, particularly before the condition was formally described and before ablation techniques were established as effective therapeutic tools.^81^ The mechanisms underlying PVC-CM remain incompletely understood, though several theories have been proposed. These include electromechanical dyssynchrony, extrasystolic potentiation, interpolation, R-R variability, and myocardial remodelling induced by short-coupled PVCs.^82^ At the cellular level, suggested mechanisms involve altered myocardial perfusion and oxygen consumption, changes in ion channel and intracellular protein expression, and modifications in autonomic tone.^68^ Dyssynchrony, in particular, has been highlighted as a possible pathophysiologic drive.^83^ Reported prevalence of PVC-CM ranges from 6–7% in populations with frequent PVCs to as high as 38% in cohorts referred for ablation.^84^

Although the body of evidence on PVC-CM and the positive effects of catheter ablation is growing, most studies linking PVCs to cardiomyopathy are retrospective or cross-sectional in design.^85,86^ A meta-analysis focusing on characteristics associated with the risk of developing PVC-CM was published in 2022.^87^ In total, 26 studies and 11 potential risk factors were included and analysed, with results indicating that eight characteristics—age, presence of symptoms, non-sustained VT, LV origin, epicardial origin, interpolation, PVC duration, and PVC burden—were significantly linked to the development of PVC-CM. Two meta-analyses examining PVC-CM and ablation outcomes were published in 2014. Zang *et al*. included data from 15 studies involving 712 patients and reported a mean PVC burden of 24% prior to ablation, along with an overall mean increase in LVEF of 7.7% following ablation (12.4% in patients with baseline LV dysfunction). These findings led to the conclusion that ablation of frequent PVCs improves cardiac function, particularly in patients with impaired LVEF. The second meta-analysis, conducted by Lamba and colleagues,^88^ focused on PVCs originating from the RVOT and analysed results from six studies. Ablation in this subgroup significantly reduced PVC burden and improved LVEF by a mean of 10.36%. Although strong evidence now supports catheter ablation as an effective intervention for improving EF in PVC-CM, some data suggest that comparable benefits may also be achieved with pharmacological therapy, provided that suppression of PVC burden is equally successful.^89^

## PVC burden, PVC morphology, and other electrophysiological features

In addition to studies evaluating the effects on cardiac function and clinical outcomes, some investigations have focused on risk stratification of patients with PVCs based on electrophysiological characteristics. Several authors have used a series of such features to construct risk scores for PVC patients.^90^ The question of whether a threshold exists in the number of PVCs has been examined in multiple studies, with inconsistent findings. Notably, a lower-than-expected PVC burden has occasionally been associated with poorer prognosis.^52,91–94^ Using receiver operating characteristic curves, Lin et al. identified 12 PVCs per day as the (surprisingly low) optimal cut-off value for increased risk of heart failure and sudden death,^51^ with sensitivity and specificity values of approximately 60%. However, other studies have failed to demonstrate a clear correlation between PVC burden and clinical outcomes.^53^

Broad PVCs, defined by the width of the QRS complex during the ectopic contraction, have been linked to adverse prognosis, with an approximate cut-off value of 150 millisecond.^95–99^ A wide QRS during PVCs may serve both as a risk marker and a risk factor: impaired myocardial conduction can reflect underlying fibrotic tissue, while slow depolarization itself can lead to dyssynchrony. Furthermore, wider PVCs may indicate an epicardial origin. PVC morphology—used as a surrogate for site of origin—has also been investigated as a tool for risk stratification. Evidence in this area appears more robust among individuals with established heart disease, in whom multifocal PVCs are associated with worse outcomes compared to unifocal.^55,100,101^ In contrast, for structurally healthy individuals with PVCs, the prognostic significance of QRS morphology remains uncertain. A notable limitation is that most relevant studies have been conducted in ablation or other settings involving highly selected patient populations.^91,96,102–106^

Other electrophysiological parameters evaluated include PVC interpolation, coupling interval, and the Peak Deflection Index^95,99,107,108^. With respect to the coupling interval, both long- and short-coupled PVCs have been reported to trigger malignant ventricular arrhythmia.^95,109,110^ This apparent discrepancy may be explained by distinct mechanisms: short coupling intervals are more likely to initiate malignant arrhythmias, whereas long intervals may increase the risk of left ventricular dysfunction. Another hypothesis suggests that pronounced rhythm irregularity itself—characterized by both long and short intervals (high ‘interval dispersion’)—serves as a predictor of cardiomyopathy.^111^

## Diagnostics and significance of imaging findings

Current guidelines recommend a comprehensive evaluation of patients with ventricular arrhythmia, including assessment of individual and family history, physical examination, 12-lead ECG, ambulatory electrocardiography, and exercise testing. Nevertheless, in clinical practice, not all patients with PVCs undergo the full range of examinations necessary to exclude structural heart disease.

An unresolved issue is whether conventional imaging techniques are sufficiently accurate to detect subtle abnormalities, such as small areas of myocardial damage that may serve as the origin of PVCs, or whether more advanced imaging modalities should be employed. CMR has gained increasing recognition in recent years as a valuable tool for identifying subtle forms of heart disease in patients with PVCs. A pioneering study by Proclemer in 1997 reported findings from CMR examinations of 19 patients with frequent monomorphic PVCs and 10 controls.^112^ The results demonstrated that RVOT dimensions were larger in PVC patients, and wall motion abnormalities were present in 16 of 19 cases, whereas all controls exhibited normal MRI findings. These observations were subsequently confirmed by several studies,^59,113–116^ some of which also established a link between CMR findings and prognostic outcomes.^117,118^ A particularly noteworthy study by Nucifora,^119^ included 46 patients with monomorphic ventricular arrhythmia of LV origin and 74 patients with idiopathic monomorphic arrhythmia of RV origin, divided into two groups. The LV group exhibited significantly more CMR abnormalities, which were also associated with adverse cardiac events. CMR has further been applied for aetiological diagnosis in populations with and without previously characterized mixed ventricular arrhythmias^120,121^ and to elucidate the differential contribution of PVCs to haemodynamic function.^122,123^ Despite these advances, most studies have employed narrow inclusion criteria regarding PVC morphology, PVC burden^124^ or other patient characteristics. Consequently, important questions remain about the broader applicability of CMR in the evaluation of PVC patients.

Although CMR is recommended in both European and American guidelines, and its role is generally more clearly highlighted in 2022 guidelines,^29^ the method is not broadly used in clinical praxis. This is partially due to its cost and limited accessibility. Echocardiography, on the other hand, is a more common evaluation method, and it has been studied whether additional echocardiographic parameters can add clinically relevant information. However, the body of literature in this field is not as large as for CMR. We have already mentioned the study by Lie about the burden of PVCs leading to PVC-CM.^92^ This study used speckle tracking echocardiography to assess left ventricular global longitudinal strain (GLS) and mechanical dispersion and stated that PVC burden correlated with GLS and mechanical dispersion but not with ejection fraction. Other studies have used speckle-tracking echocardiography to identify subtle myocardial dysfunction in PVC patients. In the study from Wijnmaalen,^125^ 49 subjects with PVCs and 26 healthy controls had normal LVEF; however, speckle tracking showed reduced ventricular strain significantly more often in PVC cases than in controls. Radial, circumferential, and longitudinal strain improved significantly after ablation but remained unchanged in untreated patients. Ling studied 40 subjects with monomorphic frequent PVCs and 40 control^126^ and detected no significant differences in standard 2D-echocardiographical findings (including EF) between the two groups; however, parameters evaluated with 3D speckle tracking echocardiography were significantly worse in the PVC group. While most studies have primarily concentrated on systolic LV function, others have adopted a broader perspective, investigating echocardiographic measures of the impact of PVCs on right ventricular, atrial, and diastolic left ventricular function,^127,128^ as well as how these parameters are influenced by successful catheter ablation.^129^

## Indications for PVC suppression

In most cases, treatment aimed at suppressing premature ventricular complexes (PVCs) is unnecessary, as PVCs are often asymptomatic or too infrequent to produce significant symptoms or adversely affect ventricular structure or function. When the daily PVC burden is low (i.e. fewer than 10 000 PVCs per day, corresponding to approximately 10% of total daily beats), therapy is generally deferred.

In patients with structurally normal hearts, the decision to treat PVCs should be guided primarily by symptom burden and, critically, by a clear correlation between symptoms and PVCs documented on ECG recordings. This correlation is essential because many patients report nonspecific symptoms, such as fatigue or reduced exercise tolerance, which may be related to PVCs due functional bradysphygmia and relative hypoperfusion, but also to other non-arrhythmia related mechanism(s). Therefore, establishing a symptom-ECG correlation helps determine whether PVCs are truly responsible for the symptoms and allows to set realistic expectations regarding the benefits of PVC suppressive therapy.

A higher PVC burden (>10–15%) has been associated with the development of left ventricular dysfunction in patients with previously structurally normal hearts, as well as with worsening ventricular function in those with underlying cardiomyopathy. Consequently, PVC suppression is generally recommended when there is clinical suspicion that frequent PVCs are causing or contributing to left ventricular dysfunction. Similarly, in patients with cardiac resynchronization therapy devices, frequent PVCs may reduce effective biventricular pacing. In such cases, PVC suppression has been shown to restore adequate pacing percentages and promote favourable left ventricular remodelling.

Finally, PVCs may serve as triggers for ventricular fibrillation, particularly when they are short-coupled (occurring at or before the peak of the T wave) or originate from the fascicular/Purkinje system. In these scenarios, interventions aimed at PVC suppression can be life-saving.

## Therapeutic approaches for PVC suppression

PVC suppression can be achieved with either pharmacologic therapy (e.g. antiarrhythmic drugs, AADs) or catheter ablation. Catheter ablation is recommended by international expert consensus documents in patients with frequent and symptomatic PVCs, whenever AADs are ineffective, not tolerated, or not preferred. In select cases where the putative site of origin of the PVC is more favourably amenable to catheter ablation, such as the right ventricular outflow tract, it is reasonable to prefer catheter ablation over AADs as a first-line therapy.

## Antiarrhythmic drugs

Clinical data on efficacy and safety of AAD therapy for PVC mostly derive from small non-controlled studies, with efficacy assessed with inconsistent and limited follow-up ECG monitoring. Beta blockers are often used as first-line therapy for symptomatic PVCs, although evidence of efficacy is scant with studies documenting clinically significant PVC count reduction in less than 25% of patients.^130,131^ Similarly, non-dihydropyridine calcium channel blockers are widely used for PVC suppression in patients with structurally normal heart, although data on efficacy of these agents are mostly limited to PVCs of fascicular/Purkinje origin.^132,133^ However, given the significant negative inotropic effect of non-dihydropyridine calcium channel blockers, these agents are avoided in patients with LV dysfunction. Class IC AADs, and particularly flecainide, are among the most effective AADs to suppress PVCs with demonstrated efficacy in reducing PVC burden in patients with and without underlying cardiomyopathy.^89,134^ However, particular caution should be exercised when prescribing these agents in patients with underlying cardiomyopathic substrates and left ventricular dysfunction given the documented risk of pro-arrhythmia and the associated negative inotropic effect that can worsen underlying heart failure.^135,136^

Class III AADs (amiodarone, sotalol) have been formally tested in randomized controlled trials for prevention of sustained ventricular arrhythmias in patients with structural heart disease,^137,138^ but not specifically for PVC suppression. Considering the potential for significant side effects associated with the use of class III AADs,^139^ the use of these agents should be mostly reserved for patients with underlying cardiomyopathic substrates and frequent PVCs for which catheter ablation may be associated with significant risks, such as PVCs originating adjacent to the proximal conduction system (e.g. para-Hisian origin) or major coronary arteries (e.g. septal LV summit region), or in patients with high burden of multifocal PVCs without a predominant morphology amenable to mapping and ablation.

## Catheter ablation of PVCs: general considerations

The ideal candidates for catheter ablation of PVCs are those with frequent ventricular ectopy from a single predominant focus.^140,141^ In these cases, it can be reasonably expected to be able to localize with high accuracy the site of origin of PVCs with mapping, and a meaningful burden reduction (or complete abolition) of PVCs following catheter ablation can be anticipated. In patients with high burden of multifocal PVCs, it is important to identify whether there are dominant (i.e. frequent and monomorphic) morphologies that may be amenable to mapping and ablation; in the absence of a clear dominant PVC morphology or cluster of morphologies that may originate from a single distinct anatomical site(e.g. papillary muscle, interventricular septum—see also below), mapping and ablation is challenging and AADs are the most appropriate strategy to suppress PVCs.^140,141^ In this context, in patients with PVCs who are being considered for catheter ablation, obtaining a 12-lead ECG of the PVCs is of crucial importance owing to its high spatial accuracy in determining the PVC site of origin, particularly in patients with structurally normal heart. Several ECG algorithms have been described to aid preprocedural localization of PVCs,^142–145^ with some involving relatively cumbersome ECG measurements. Previous publications have provided excellent ECG-examples that can be used in this process.^146^ From a practical standpoint, the most relevant ECG characteristics to guide procedural approaches and anticipate mapping/ablation complexity are the following (*Figure 2*):

**Figure 2:**
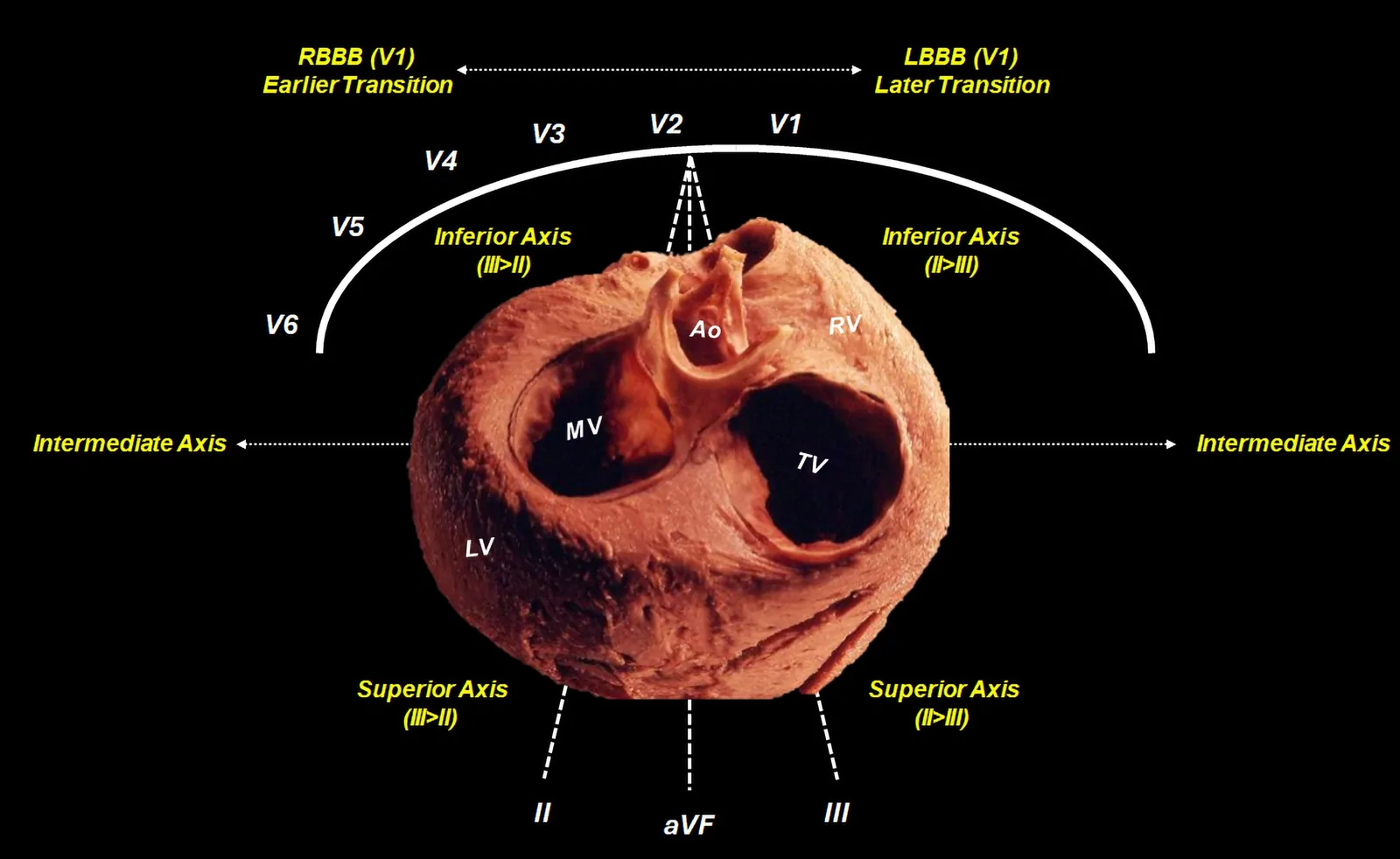
Schematic representation of most common perivalvular PVC ECG patterns. Outflow tract PVCs display an inferior axis (RVOT typically more positive in lead II than III; LVOT typically more positive in lead III than II) with LBBB (RVOT or septal LVOT origin) or RBBB (lateral LVOT and mitral annular locations). Perimitral and peritricuspid PVCs may have variable frontal plane axis depending on the specific sites of origin around the annulus. Heart image courtesy of the UCLA Cardiac Arrhythmia Center, Amara-Yad Project Collection.

ECG pattern in lead V1. This identifies the ‘bundle branch block’ pattern and the general origin from the left ventricle (i.e. right bundle branch block—RBBB—pattern) or right ventricle/interventricular septum (i.e. left bundle branch block—LBBB—pattern).

The QRS axis in the frontal plane. An inferior QRS axis (i.e. predominantly positive forces in the inferior leads II, III, aVF) indicates an origin from the superior base of the heart (i.e. outflow tract or superior aspect of atrioventricular valves). On the other hand, a superior QRS axis (i.e. predominantly negative forces in the inferior leads) indicates origin from the diaphragmatic aspect of the ventricles. Furthermore, the axis deviation (i.e. right vs. left) corroborates origin from the left ventricle (right axis) vs. right ventricle (left axis).

The precordial QRS transition zone is helpful in determining basal vs. apical origin. In RBBB pattern PVCs, a progressively later transition zone indicates more basal ventricular origin. In LBBB pattern PVCs, a later transition zone indicates origin towards the free wall of the RV whereas more septal origin are associated with progressively earlier transition zones.

Total QRS width: septal sites of origin have narrower QRS widths compared to free wall sites.

Noninvasive ECG imaging tools have been developed with the main aim of improving preprocedural recognition of the putative site of origin of focal arrhythmias, including PVCs. While some systems use information from the 12-lead ECG to project on a three-dimensional heart model the putative PVC site of origin based on automated vector analysis (Vektor Medical, San Diego, California),^147^ other systems derive information from dedicated multielectrode vests applied to the patient’s torso to project the local activation onto a three-dimensional geometry of the heart obtained with preacquired chest computer tomography.^148^ To date, the real clinical advantage of these more advanced noninvasive ECG imaging solutions over information derived from analysis of 12-lead ECG utilizing established criteria is largely unproven.

Invasive cardiac mapping remains the most accurate method to identify the PVC site of origin. In general, invasive mapping consists of two main approaches, namely: activation mapping and pace-mapping. Activation mapping is the preferred technique, but it requires PVCs to have a sufficient intraprocedural burden to allow for adequate localization.^149^ The main goal of activation mapping is to identify the intracardiac earliest activation site in relation to the onset of the PVC QRS; this requires point-by-point analysis of the local activation time with rowing intracardiac catheters. Currently, multipolar large-footprint catheters incorporating multiple small electrodes with tight interelectrode space and covering a larger surface area compared to conventional small-tip mapping and radiofrequency ablation catheters represent the gold standard technology for identification of near-field cardiac activation, although obtaining adequate contact with cardiac regions that are commonly implicated in idiopathic PVCs such as the perivalvular myocardium or intracavitary papillary muscles may be challenging.

For patients with infrequent PVCs and to further corroborate findings from activation mapping, pace-mapping is commonly utilized, with the aim of reproducing the exact 12-lead ECG morphology of the target PVC by pacing from different cardiac sites with a rowing catheter.^150,151^ Current generation mapping software is able to automatically compare the 12-lead ECG of spontaneous PVCs with QRS derived from pace-mapping creating a ‘match’ score. Typically, a score >0.9 (i.e. >90% QRS pattern match) is considered a good pace match.

## Common sites of origin of PVCs: implications for mapping and ablation

PVC sites of origin tend to cluster to specific cardiac regions, such as the perivalvular myocardial tissue (e.g. outflow tract in proximity to the aortic/pulmonary valves or perimitral/peritricuspid valve), intracavitary structures such as the papillary muscles and moderator band, and, more rarely, from the Purkinje network. With the exception of PVCs from the Purkinje network and intracavitary structures which are, by definition, targeted only with an endocardial approach, perivalvular PVCs may require endocardial, epicardial or direct intramural mapping (via venous or arterial septal perforators, when available) depending on their site of origin.

### Outflow tract PVCs

The ventricular outflow tracts are the most common sites of origin of idiopathic PVCs.^141,152^ Historically, the right ventricular outflow tract (RVOT) has been reported as the most common site of origin of idiopathic PVCs,^153^ although more recent case series have documented an increase in prevalence of left ventricular outflow tract (LVOT) PVCs.^152^ As septal outflow tract PVCs are by far the most common type of PVCs referred for catheter ablation,^154^ the reported increase in prevalence of LVOT PVCs may reflect an increased awareness of the importance of systematic mapping of both right and left outflow tracts, with resulting improved intraprocedural identification of LVOT sources.

Outflow tract PVCs are invariably characterized by an inferior QRS axis—analysis of the relative forces in leads II and III is helpful to distinguish right (R wave in II greater than III) vs. left (R wave in III greater than II) outflow tract origin. As aforementioned, QRS width and transition zone are also helpful to distinguish between different sites around the right and left ventricular outflow tracts, with wider QRS durations indicating non-septal origin, and an RBBB pattern or LBBB with early (before V4) transition more typical of LVOT origin. Unless ECG criteria clearly indicate an LVOT focus, intraprocedural activation mapping is typically carried out sequentially from the RVOT and moving to the LVOT in case of early septal activation, particularly in the presence of a broad area of similarly early activation (*Figure 3*). Pace-mapping is mostly useful for non-septal outflow tract locations, although the presence of preferential conduction within the outflow tract, together with the suboptimal spatial resolution of 12-lead ECG, should be taken into consideration when using pace-mapping to localize the putative site of origin of outflow tract PVCs.^155,156^

**Figure 3:**
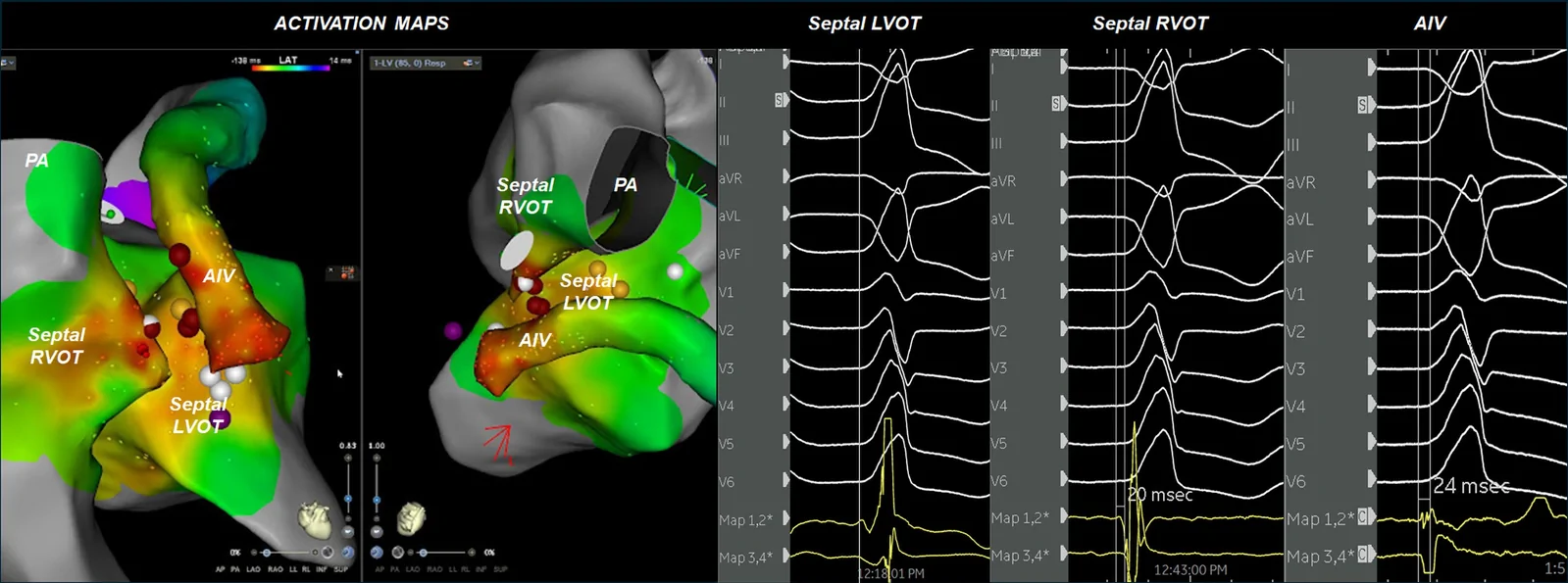
Example of multi-site mapping of septal outflow tract PVC. Multiple sites at the septal RVOT, septal LVOT, and epicardially via the anterior interventricular vein (AIV) were mapped and ablation was delivered to all three sites to achieve durable PVC suppression.

The outcomes of catheter ablation of outflow tract PVCs are generally good, but success rates are variable, which reflects the anatomical complexity of this region. For classical outflow tract VAs accessible with an endocardial approach, such as those from the endocardial outflow tract (RVOT or LVOT) or those easily accessible from the aortic/pulmonary cusp region, ablation outcomes have been reported as excellent (>90% efficacy in PVC suppression).^131,157^ One important safety consideration when targeting outflow tract PVCs from the aortic or pulmonic cusp region is the potential proximity to the proximal coronary arteries. Intraprocedural imaging with intracardiac echocardiography (ICE) has been shown to be very reliable in localizing the position of the mapping/ablation catheter in relationship to the proximal coronary arteries,^158^ thus preventing the need for intraprocedural angiography. However, in centres where ICE is not available or if the operator is not proficient with the use of ICE to localize the proximal coronary arteries, intraprocedural coronary angiography should be considered when targeting outflow tract arrhythmias from the aortic or pulmonary cusp region.

An important subset of outflow tract PVCs may have an intramural site of origin.^159–162^ Indirect clues for intramural PVC origin include a similar broad area of early activation on both sides of the interventricular septum (or endo-epicardially), lack of a distinct QS unipolar electrogram pattern at the earliest endocardial or epicardial site, and lack of good pace-match from accessible endocardial or epicardial sites.^160,161^ Whenever possible, direct intramural mapping via accessible septal perforators of the venous (preferred approach) or arterial system can confirm an intramural origin by proving earliest intramural activation.^159^ In most of these cases, catheter ablation can be performed from anatomically adjacent endocardial (or aortic/pulmonary cusp) sites provided that the anatomical distance between the earliest intramural site and the adjacent endocardial site is within 8–10 mm.^159,163,164^ If conventional unipolar radiofrequency ablation fails, bailout strategies such as bipolar radiofrequency ablation, irrigated ablation utilizing a lowered ionic irrigant (i.e. half-normal saline), ethanol ablation from within the septal perforators (*Figure 4*) or, more recently, pulsed field ablation (PFA) have been used to increase lesion depth and enhance procedural success.^165–167^

**Figure 4:**
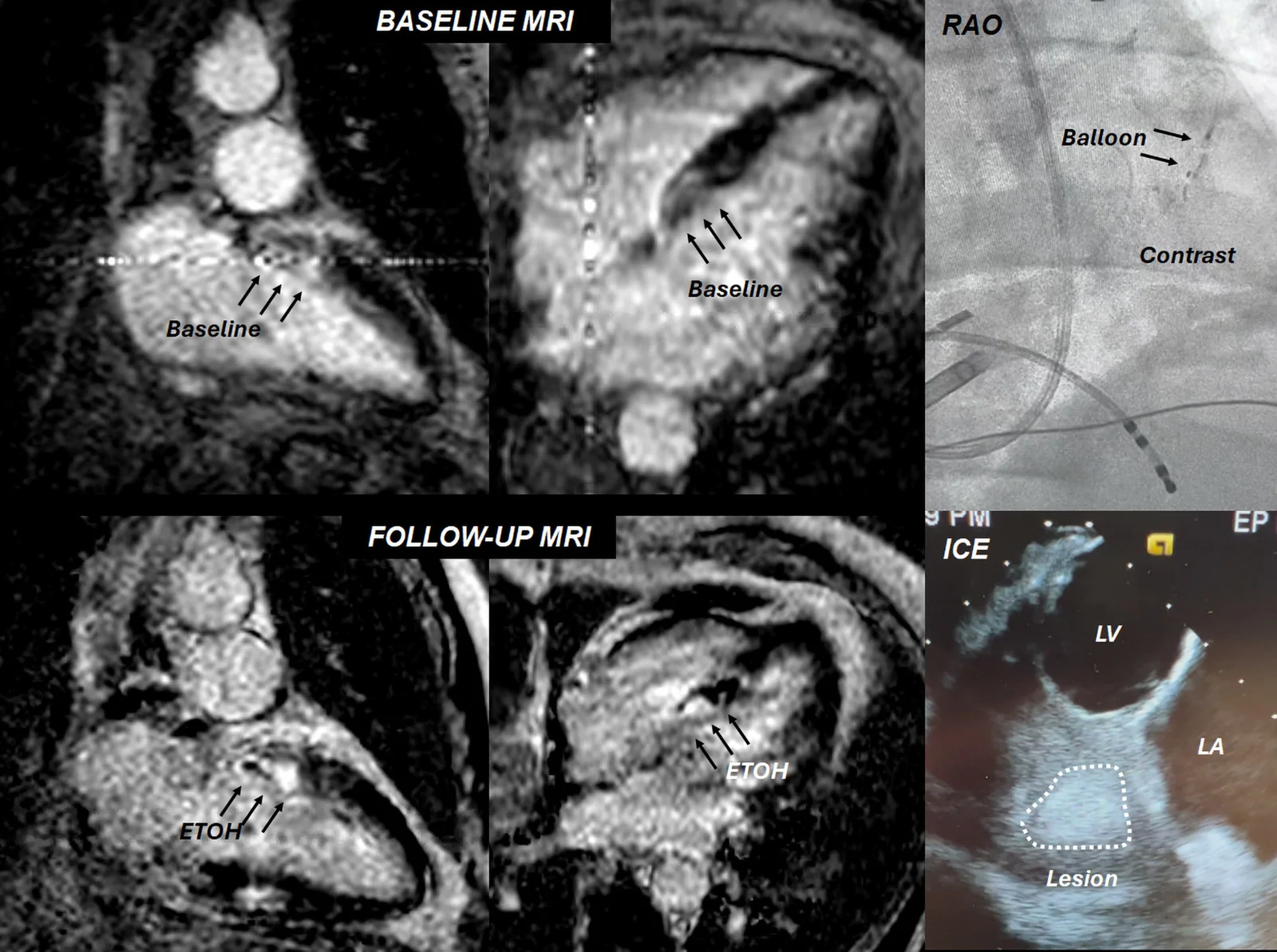
Ethanol ablation following balloon occlusion of the first venous septal perforator of the anterior interventricular vein to target an intramural PVC in a patient with non-ischaemic cardiomyopathy and basal antero-septal substrate demonstrated on MRI. Note the acute echogenic ablation lesion visible on ICE (bottom right panel) following ethanol injection. Repeat MRI shows effective intramural chemical ablation.

### Perimitral and peritricuspid PVCs

The myocardial tissue at the perimitral or peritricuspid ventricular ostia is also a common site of origin of idiopathic PVCs. Perimitral PVCs have a typical RBBB pattern (unless arising from the most septal aspect of the annulus where they may also display a LBBB pattern) and right axis deviation, whereas peritricuspid PVCs always display a LBBB pattern and left axis deviation. The polarity of the QRS complexes in the frontal plane depends on the PVC site of origin along the mitral or tricuspid annuli (i.e. PVCs from the superior portion of the mitral/tricuspid annulus will have an inferior axis, whereas PVCs from the inferior portion of the mitral/tricuspid annulus will have a superior axis). Unlike outflow tract PVCs, which are typically observed in patients with no underlying structural heart disease, a subset of patients with perimitral or peritricuspid PVCs may have an associated concealed cardiomyopathy, especially if PVCs are multifocal.^168,169^ Mitral annular PVCs most commonly arise from the superior portion of the mitral annulus,^170^ and, similarly to outflow tract PVCs, may originate from the subendocardium, epicardium, or mid-myocardium. Mapping is usually performed from the endocardium and/or epicardium via the coronary sinus, as direct percutaneous epicardial mapping has a low yield due to the presence of coronary arteries and epicardial fat around the atrioventricular groove. Peritricuspid PVCs account for up to 8% of idiopathic PVCs, and most commonly arise from the septal aspect of the annulus.^171^ Catheter stability during mapping and ablation of the ventricular aspect of the tricuspid annulus can be challenging owing to the beat-to-beat anteroposterior motion of the annulus together with increased susceptibility to respiratory catheter drift. In general, the success rate of catheter ablation of free wall peritricuspid PVCs has been reported higher compared to septal locations,^171^ possibly related to the frequent intramural origin and/or proximity to the proximal conduction system (i.e. para-Hisian location, see also below) of some septal peritricuspid PVCs.

### PVCs from intracavitary structures

Intracavitary structures, including the left and right ventricular papillary muscles and the moderator band, are a recognized source of idiopathic PVCs, although they occur far less commonly than perivalvular PVCs.^172–175^ In contrast, among patients with PVC-triggered ventricular fibrillation, intracavitary sites and the Purkinje network represent some of the most frequent sites of origin.^176,177^ Papillary Muscle PVCs tend to have a focal, non-reentrant mechanism tied to abnormal automaticity or triggered activity. Rather than being induced by programmed stimulation, they are generally exercise-related and brought on by catecholamine surges.^178^

In the first description by the University of Alabama group in 2008, out of 290 consecutive patients referred for catheter ablation of idiopathic PVCs or VT, 7 (2.4%) were identified with a site of origin at the postero-medial papillary muscle (PM-PM).^173^ The same group subsequently described another series of six patients with focal ventricular arrhythmias where the site of origin was identified at the anterolateral papillary muscle (AL-PM).^175^ Following this initial description, ventricular arrhythmias from intracavitary structures have been increasingly recognized and described by other groups,^170,172,174,179^ including right ventricular papillary muscles and moderator band sources.^180,181^ LV papillary muscle PVCs have a characteristic RBBB pattern with relatively short intrinsicoid deflection time (<74 ms) and mid-wall transition (V3-V4).^182^ The frontal plane axis depends on the origin: PM-PM typically displays a left superior axis, whereas AL-PM displays a right inferior axis with characteristic inferior lead ‘discordance’ (i.e. negative in lead II, positive in lead III). RV papillary muscle and moderator band sources have a LBBB pattern with a typical left superior axis (more negative in III than II) and late (>V5) transition.^181^ Mapping and catheter ablation of PVCs originating from intracavitary structures can be challenging owing to the complex anatomy and mobile nature of the papillary muscles during the cardiac cycle. Intraprocedural ICE imaging has proven invaluable for defining the anatomy of the papillary muscles/moderator band, monitoring for catheter-tissue contact, and validating complete sampling around the complex geometry of these structures (*Figure 5*).^172,174,176,180–183^ Overall, catheter ablation of PVCs from intracavitary structures has been shown safe but less effective compared to the more typical outflow tract sites, with success rates ranging from 80% to 92% and more frequent need for multiple ablation procedures.^184^ This is largely related to inability to deliver adequate radiofrequency lesions at the target site due to challenges in obtaining and maintaining adequate tissue-catheter contact—in this context, the use of contact force sensing catheter technologies is helpful to validate adequate contact. In order to overcome these limitations, cryoablation has been utilized successfully in cases where radiofrequency has failed.^185–187^ One important advantage of cryoablation over radiofrequency ablation for intracavitary structures is the tissue-catheter adherence and, hence, stability after reaching cryoablation temperatures (<−70°C).^185–187^

**Figure 5:**
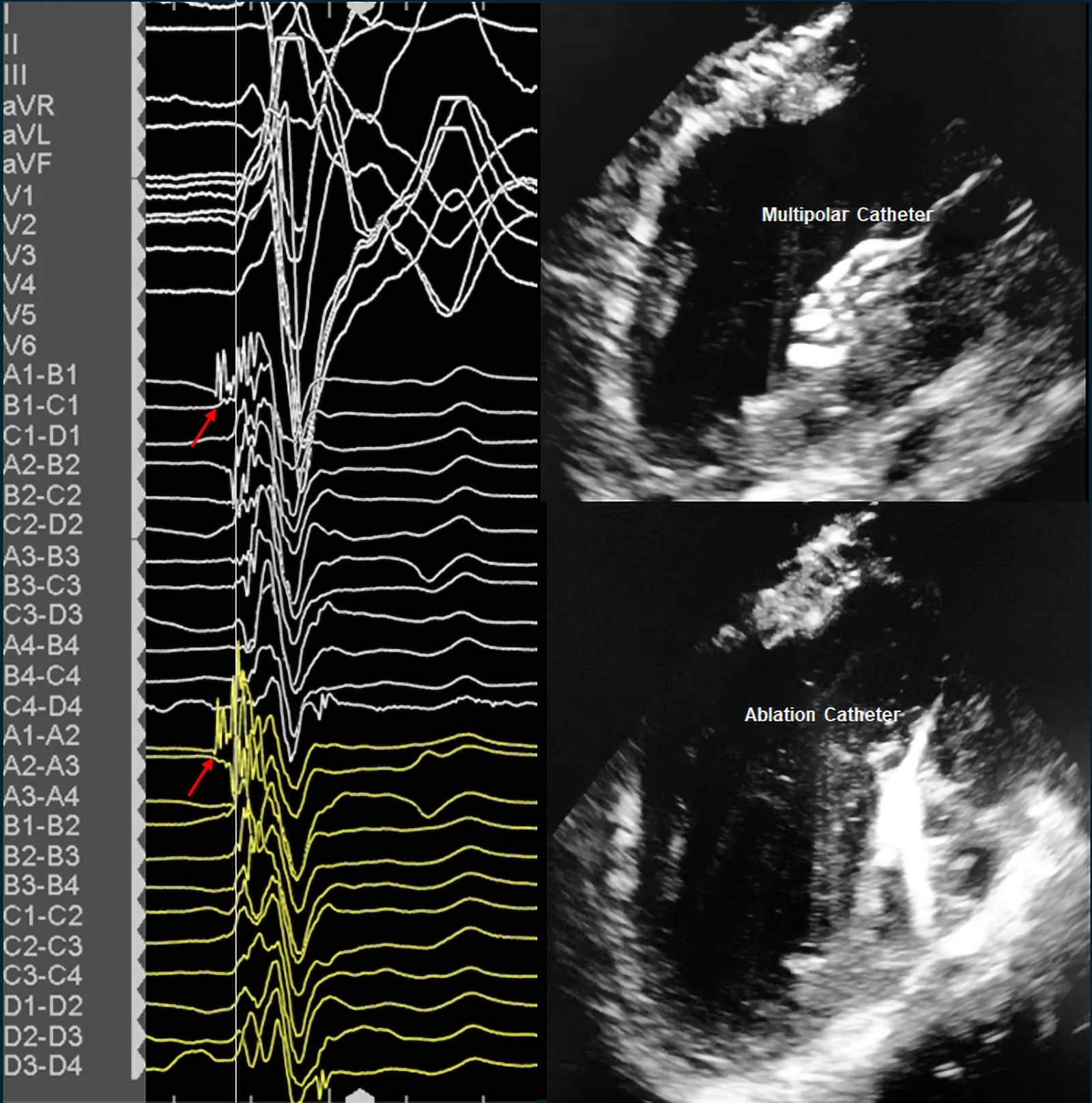
Mapping and ablation of PVC arising from the postero-medial papillary muscle of the left ventricle. Local activation shows sharp pre-QRS potentials (30 ms preceding the QRS complex) on the body of the papillary muscle as validated with ICE. Ablation results in suppression of the PVC.

### PVCs from epicardial locations

Approximately 4–10% of patients with idiopathic PVCs have epicardial sites of origin,^152,188^ with the most common site being the perivalvular LVOT epicardium, a region commonly referred to as the LV summit being the most superior aspect of the LV epicardium.^189–191^ The LV summit is bordered by the bifurcation of the left anterior descending (LAD) and left circumflex (LCx) coronary arteries, and laterally crossed by the great cardiac vein (GCV) at its junction with the anterior interventricular vein (AIV) (*Figure 5*).^189–191^ The course of the GCV divides the LV summit into two regions: a medial, more superior area above the GCV that is generally not amenable to direct catheter ablation because of its close proximity to major coronary arteries and the presence of thick epicardial fat (the ‘inaccessible’ region), and a more lateral, inferior area below the GCV that may be directly reachable with ablation because it lies farther from the main coronary vessels (the ‘accessible’ region). Several ECG markers have been proposed to identify PVCs arising from the LV summit and to help distinguish whether the focus originates from the accessible or inaccessible portion, which can guide procedural strategy.^189,192^ LV summit arrhythmias display either a RBBB pattern with positive concordance across the precordial leads, or a LBBB pattern with an early (≤V2) precordial transition with characteristic right inferior axis. Additional ECG clues to epicardial origin include slurring at the onset of the QRS complex which reflects slow epicardial-to-endocardial conduction and this can be quantified using the following criteria: (i) pseudo-delta wave (time to earliest rapid deflection in the precordial leads) ≥34 ms, (ii) intrinsicoid deflection time in V2 (interval to peak R wave) ≥85 ms, (iii) maximum deflection index (MDI: shortest interval to maximal deflection divided by QRS duration) ≥0.55, and (iv) shortest RS complex (time to earliest QRS nadir in precordial leads) ≥121 ms.^189,191,193^ Anatomically opposite to the LV summit is the ‘cardiac crux’, which is the most posterior and inferior epicardial region of the LV adjacent to the epicardial RV inferior base (*Figure 5*). In this region, the posterior atrioventricular and interventricular grooves meet near the origin of the middle cardiac vein (MCV) from the coronary sinus (CS). Idiopathic PVCs from the crux/MCV typically show an LBBB pattern with transition ≤V2, a left superior axis with QS and slurred inferior-lead deflections, and an MDI ≥0.55. Procedurally, the crux can be divided into a basal portion (often ablated from the MCV or proximal CS) and an apical portion (which may require subxiphoid epicardial access).^194^

Catheter ablation of epicardial PVCs is challenging as direct ablation at the earliest site of origin is often not possible due to proximity to major coronary arteries or presence of epicardial fat, as aforementioned.^164,189,194^ An anatomical approach targeting adjacent and accessible vantage points is typically adopted to target these arrhythmias, with variable success rates (ranging from 50% to 80%) largely depending on the anatomical distance between the epicardial site of origin and the adjacent target site.^164,190,191^ For LV summit arrhythmias, common adjacent mapping and ablation vantage points include the coronary cusp region, the LVOT perivalvular endocardium, the antero-septal RVOT and the distal GCV/AIV. An anatomical ablation approach is most effective when adjacent targets are ≤12.8 mm apart or when their local activation times differ by ≤7 ms.^164,195,196^ Prolonged radiofrequency applications (3–5 min) have with gradual power escalation up to 40 Watts have been associated with improved long-term PVC suppression.^197^

### PVCs linked to the conduction system (para-Hisian, Purkinje-related)

Between 3% and 9% of idiopathic PVCs originate in the basal upper septum in proximity to the His bundle region.^198,199^ Para-Hisian PVCs have specific ECG features including a LBBB pattern with QS pattern in V1 and early precordial transition (V2-V3), narrow QRS duration indicating septal origin, monophasic R waves in lead I reflecting right-to-left LV activation, and a characteristic ‘inferior lead discordance’ with predominant positive (or more positive) forces in lead II and more negative in lead III.^198–200^ As catheter ablation at the earliest Para-Hisian site is associated with a substantial risk of collateral injury to the conduction system, these arrhythmias are best approached from anatomically adjacent vantage points such as the posterior coronary cusp region (right coronary cusp and its junction with the non-coronary cusp).^198^ When ablation from these sites is unsuccessful, direct ablation at the earliest site can be considered, although the risk of periprocedural injury to the conduction system may be significant in these cases. Purkinje-related PVCs originate from ramifications of the right and left bundle branches, and are commonly implicated in VF triggers both in patients with structurally normal hearts and in patients with underlying cardiomyopathic states.^201–203^ ECG morphologies associated with Purkinje PVCs typically replicate focal antegrade activation along the fascicle of origin (although different degrees of fusion due to retrograde invasion of the other fascicles is possible), with PVCs originating from the RBB and its ramification displaying an LBBB morphology and PVCs originating from the LBB a RBBB morphology with either right inferior (left anterior fascicle origin) or left superior (left posterior fascicle origin) axis. Catheter ablation targeting the earliest Purkinje potentials has been shown to be highly effective in suppressing Purkinje-related PVCs,^201^ and abolishing episodes of recurrent VF in up to 80% of cases.^204^

## Catheter ablation energy sources for PVCs: current standards and future directions

Open irrigated radiofrequency ablation remains the current standard of care for targeting idiopathic PVCs, with an excellent track record of safety and efficacy. As aforementioned, lesion depth with conventional small-tip irrigated RF remains its main limitation, particularly when targeting intramural foci or mobile structures where tissue-catheter contact is poor and/or intermittent. Bailout ablation strategies have been described with the main purpose of increasing the lesion depth in challenging PVC locations. These include direct intramural tissue chemical ablation with alcohol injection within a venous or arterial septal perforator, bipolar radiofrequency ablation, ablation utilizing lower ionic irrigation, cryoablation (for intracavitary structures), and pulsed field ablation (PFA).^159,165,167,205^ The latter represent a particularly promising technology given the more selective nonthermal ablation with potential for lower risk of collateral damage to adjacent tissues.^206,207^ Additional advantages include better lesion depth and penetration through scar as compared to radiofrequency ablation.^208^ In a randomized preclinical study,^209^ Younis *et al*. compared contact force sensing open irrigated radiofrequency ablation with monopolar PFA in common idiopathic PVC locations including the LVS region (targeted from the distal GCV/AIV), the LV papillary muscles and direct epicardial ablation (75% of cases with interposed epicardial fat). Overall, PFA outperformed radiofrequency ablation lesion depth in all tested locations,^209^ suggesting that this ablation technology may offer real additive advantages over radiofrequency ablation for challenging idiopathic PVC sites. On the other hand, the risk of PFA-induced coronary vasospasm when targeting sites adjacent to major coronary arteries remains a concern for perivalvular PVC ablation.^210,211^ Thus far, the clinical experience with PFA for idiopathic PVC ablation is still early and limited to small case series. Additional investigation is needed to determine the role of PFA for idiopathic PVC ablation. In particular, prospective multicentre studies and randomized controlled trials comparing PFA with RF are warranted. Beyond acute procedural success, future studies should evaluate long-term arrhythmia-free survival, lesion durability, procedural efficiency, quality-of-life outcomes, and the impact on ventricular function. Furthermore, as lesion characteristics and safety profiles may vary according to PVC origin and anatomical substrate, dedicated studies are needed across different ventricular locations. Such investigations will be essential to define the optimal indications, safety, efficacy, and potential advantages of PFA within the evolving landscape of PVC ablation.

In the management of refractory PVCs, several advanced strategies exist beyond standard RF ablation. For papillary muscle PVCs, cryoablation provides better catheter stability, translating into higher rates of acute success and fewer recurrences compared to RF.^186^ When foci are intramural, deeper lesions can be achieved through approaches such as bipolar ablation^212^ needle ablation, or irrigation with half-normal saline.^167^

## Conclusions and future perspectives

PVCs are so commonly encountered that they have been defined as ‘ubiquitous’^213^ and even as ‘the incidentalomas of cardiac electrophysiology’.^214^ Despite their frequency, their prognostic significance, associated morbidity, and optimal management remain subjects of ongoing debate. Because PVCs can appear in a wide range of individuals—including older patients with cardiac or noncardiac comorbidities, many of whom are asymptomatic—any decision to pursue further diagnostic evaluation must be guided by general medical and ethical principles, with the goal of determining whether additional testing is likely to benefit the patient. When a diagnostic work–up is undertaken, its primary objective should be to identify or exclude underlying structural heart disease. In patients without structural abnormalities, management should focus on assessing future risk, considering PVC burden, morphology, and other electrocardiographic characteristics. Cardiac magnetic resonance (CMR) imaging, particularly the detection of myocardial fibrosis, has demonstrated prognostic value. Treatment of PVCs is typically symptom–driven, yet therapy may also be considered in asymptomatic individuals when reduction of PVC burden is desirable. Catheter ablation is generally more effective than pharmacologic therapy for PVC suppression.

In recent years, new technologies for rhythm monitoring have emerged. Smartphones and smartwatches can now detect PVCs using photoplethysmography–based algorithms^215^ a development that will likely increase the identification of incidental PVCs. This trend underscores the need for evidence–based decision–making to avoid unnecessary interventions. At the same time, ensuring access to advanced diagnostic and therapeutic tools—such as CMR, genetic testing, and invasive electrophysiologic studies—remains essential for the appropriate application of current scientific knowledge.
